# Control of Neuronal Network in *Caenorhabditis elegans*

**DOI:** 10.1371/journal.pone.0139204

**Published:** 2015-09-28

**Authors:** Rahul Badhwar, Ganesh Bagler

**Affiliations:** Centre for Biologically Inspired System Science, Indian Institute of Technology Jodhpur, Jodhpur, Rajasthan, India; CSIR-Central Drug Research Institute, INDIA

## Abstract

*Caenorhabditis elegans*, a soil dwelling nematode, is evolutionarily rudimentary and contains only ∼ 300 neurons which are connected to each other via chemical synapses and gap junctions. This structural connectivity can be perceived as nodes and edges of a graph. Controlling complex networked systems (such as nervous system) has been an area of excitement for mankind. Various methods have been developed to identify specific brain regions, which when controlled by external input can lead to achievement of control over the state of the system. But in case of neuronal connectivity network the properties of neurons identified as driver nodes is of much importance because nervous system can produce a variety of states (behaviour of the animal). Hence to gain insight on the type of control achieved in nervous system we implemented the notion of structural control from graph theory to *C. elegans* neuronal network. We identified ‘driver neurons’ which can provide full control over the network. We studied phenotypic properties of these neurons which are referred to as ‘phenoframe’ as well as the ‘genoframe’ which represents their genetic correlates. We find that the driver neurons are primarily motor neurons located in the ventral nerve cord and contribute to biological reproduction of the animal. Identification of driver neurons and its characterization adds a new dimension in controllability of *C. elegans* neuronal network. This study suggests the importance of driver neurons and their utility to control the behaviour of the organism.

## Introduction

Control of complex networks is an emerging topic in the areas of network science. One such example network in which control of physiological activities/state of the network is of crucial importance is that of neuronal connectivity network. Controllability naturally raises two key questions: what are the points of control and what is to be controlled. Determination of such points of control can be achieved with the help of various graph theoretical measures such as degree, betweenness centrality, closeness and using importance of nodes identified by evolutionary algorithm [1]. The idea of control of brain states is aligned with the studies on control of behaviour (state) of an organism by identifying and controlling a few important regions (nodes) via external inputs (impulses of electric or magnetic fields). From a connectionist paradigm, brain could be thought of as a network of neurons, a complex dynamical system, the state of which is to be controlled. This aspect has been studied as ‘structural control’ in a network aimed to be achieved with the help of a few ‘driver nodes/neurons’.

**Fig 1:**
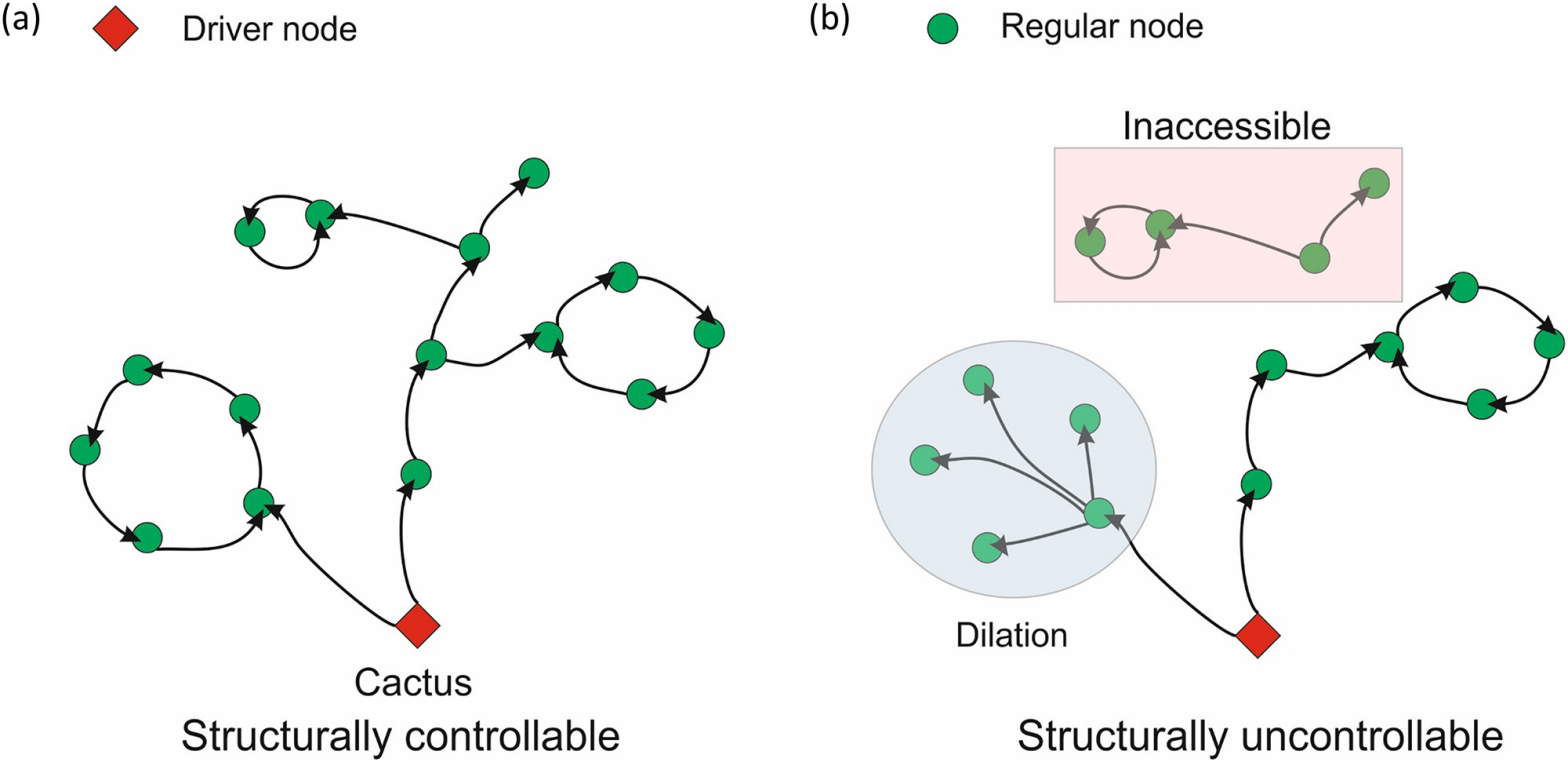
(a) Structurally controllable cacti structure where the driver node is represented in red. (b) Presence of dilations and inaccessible nodes makes the network structurally uncontrollable.

It has been proposed that networks possessing cacti structure (without having inaccessible nodes or dilations) are controllable as shown in Fig 1 [2]. A structural network with linear time invariant dynamical system could be represented as Eq (1), where *x*(*t*) represents the state of the system at time *t*, *A* is the state matrix, *B* input matrix and *u*(*t*) is input signal.

$$\begin{array}{c} x(t)=Ax(t)+Bu(t) \end{array}$$ (1)

The state of such a system is proven to be controllable only if it possess full rank [3, 4].

### Structural controllability

Finding full rank of the network for structural controllability is a computationally expensive task requiring a brute force search. A bypass method of maximal matching can perform the task efficiently by finding unmatched nodes that are known as driver nodes [5] with an algorithmic complexity of $O(\sqrt{N}\times E)$ where *N*&*E* denotes the number of nodes and edges respectively. Matched nodes are the ones which share the link in a maximal matching, else they are unmatched. These unmatched nodes are of importance because these have directed paths to matched nodes allowing full controllability of the network. The driver nodes are known to avoids hubs which are essential to maintain network integrity [5]. Different algorithms, with varying complexity, have been proposed for finding maximal matching in a network [6–10]. Among them Hopcroft-Karp has the maximum flexibility and least complexity [7]. For neuro-biological systems we found critical neurons, termed here as driver neurons (*D*_*n*_), that fall into the category of unmatched nodes using maximal matching criterion. Albeit, what is exactly controlled by the driver neurons is not very clear. To find out the contributing factors of controllability in neuro-biological system one requires prior knowledge of network structural architecture.

### *C. elegans* neuronal network

**Fig 2:**
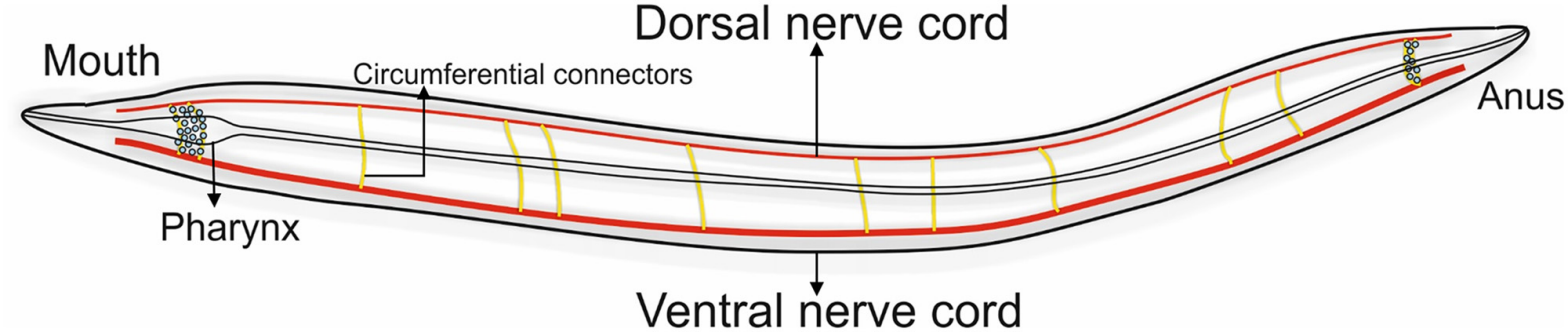
Diagrammatic representation of *C. elegans* nervous system. The gastrointestinal tract lies in the middle of the body. Pharyngeal and circumferential ring neurons (yellow) are responsible for communication between dorsal nerve cord and ventral nerve cord.

*Caenorhabditis elegans* (*C. elegans*), a nematode, is a model biological organism whose neuronal network is fully charted [11]. This hermaphrodite animal has rudimentary nervous system consisting of 302 neurons and is able to process complex information of senses, behaviour and even memory [12]. The neurons are divided into various subtypes and are classified based on their functional roles, location within the body of the animal and span of the neuron axons. According to functional roles, neurons are primarily of three types viz. sensory neurons, motor neurons and inter neurons. Sensory neurons pick up external signals to which the animal responds by sending motor signals to effector organs through motor neurons which connect to command inter-neurons on dendritic side and neuro-muscular junction on the axonal side [13]. Motor neurons are distributed mainly over the ventral nerve cord (VNC) with ganglia at each end [14] some of which extend their processes circumferentially to form a dorsal nerve cord (DNC)as shown in Fig 2. Both VNC and DNC control locomotion of the animal.

In accordance with definition of driver nodes, these critical neurons control the state of neuronal network when provided with external input. To investigate this state space and what kind of changes one can bring by controlling *D*_*n*_ in *C. elegans* state we examined phenotypic properties of these neurons. Study of properties such as location, functional type and span of neurons provided us with the potential functional association of driver neurons. Further we investigated specific biological functions underlying these neurons with the help of gene ontological enrichment studies.

### Gene ontology

The functional association of gene products with the help of gene ontological studies can provide an insight to three biological domains: (a) biological processes, (b) cellular components and (c) molecular functions. Gene ontology (GO) enrichment process provides biological functions associated with the expression profile of a particular set of genes in the background of all the genes which are expressed in the organism. We performed a group level analysis using gene ontology enrichment to associate *D*_*n*_ expression profile to major biological functions.

## Materials and Methods

### *C. elegans* neuronal network

*C. elegans* neuronal connectivity data was obtained from WormAtlas (www.wormatlas.org) [15]. The data comprised of 297 neurons and 2345 synaptic connections. This represents an unweighted network where multiple synaptic contacts/connections between neurons were merged. Neuromuscular junctions were excluded from the data. Neuronal connectivity data were represented as a directed unweighted graph, where neurons represent nodes and synaptic connections represent links.

### Identification of driver neurons

Neurons that are critical for controlling the dynamical state of the network by providing an external input are termed as ‘Driver Neurons (*D*_*n*_)’. Driver neurons (nodes) in a directed graph could be identified as ‘unmatched nodes’. A node is unmatched if the maximum set of links that do not share start or end nodes are not pointing at it. Hopcroft-Karp algorithm for identification of maximal matching nodes was implemented to arrive at the exact set of driver neurons [7]. This algorithm computes the matching *M* in a graph *G* = (*V*,*E*), having *V* vertices and *E* edges, such that vertex *V*_*i*_ has at most one incoming edge *E*_*j*_. Matching is maximal if no other permutation of matching exists M′⊃M[16]. Thus it provides a vector *p*(*j*) = *i* if column *j* is matched to row *i*, or zero if column *j* is unmatched. These unmatched columns are then referred to as the driver neurons. Matching of this short is of the order of $O(\sqrt{N}\times E)$ complexity. The matching obtained is unique hence *D*_*n*_ were extracted without any uncertainty. This method of identification of maximal matching (driver) nodes has been successfully implemented by Lui et.al. [5].

### Characteristics of driver neurons

The neurons from *C. elegans* neuronal network were classified on the basis of their anatomical locations (head, mid and tail), span (short and long), and function (sensory, motor and interneurons). The information of neuronal locations and span were obtained from White et. al. [11], whereas that of functions were obtained from Hall and Russell [17]. Further statistics of driver neurons were obtained in terms of their phenotypic properties (location, span and function) with the aim of characterizing them. Every neuron was thus characterized in terms of its ‘phenoframe’ which refers to a composite set of its phenotypic properties.

### *C. elegans* neuronal gene co-expression network

**Fig 3:**
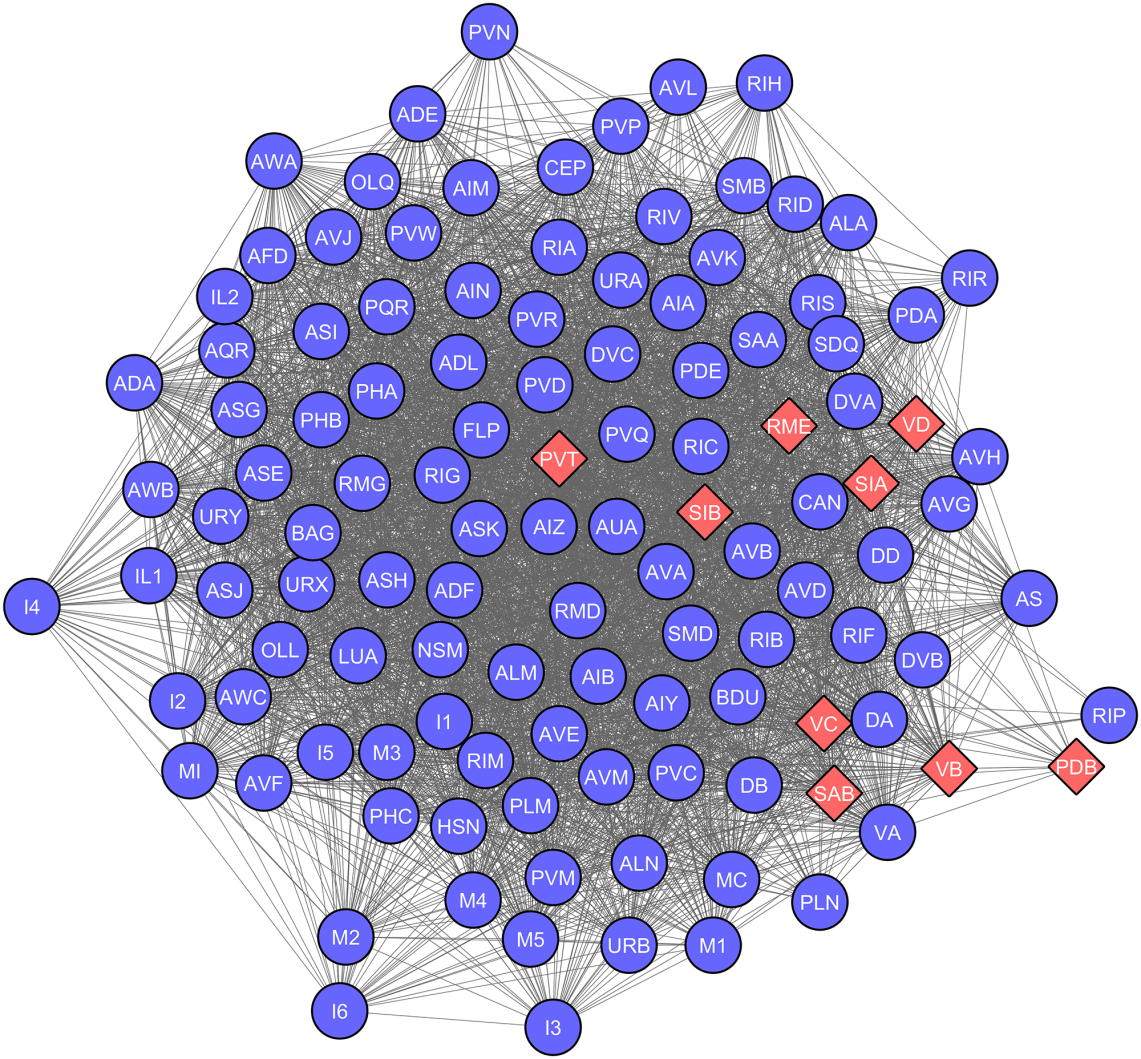
Neuronal network of *C. elegans* based on co-expression of genes (GCN). Each of the 116 neuronal groups is represented as a node. A weighted edge between any two neuronal groups represents the extent of gene co-expression. The shape and colour of the neuronal groups depict presence (Red Diamonds) or absence (Blue Circles) of driver neurons in them. The *C. elegans* co-expression based network is heterogeneous with 9 groups holding all the driver neurons, whereas rest of the 107 groups were devoid of driver neurons.

To explore genetic underpinnings of driver nodes we further created the gene co-expression network of *C. elegans* with the help of expression profiles of individual neurons. The expression data was obtained from WormWeb (www.wormweb.org), where in genes expressed in each neuron group was collected (data available in S1 Data). 297 neurons of *C. elegans* are classified into 116 groups on the basis of their expression profiles. Neurons within a group tend to have similar functional associations. *C. elegans* neurons are represented by 433 non-redundant genes, known to be expressed in neuronal cells. Towards creating a biologically relevant network of genes underlying the neuronal architecture, a bipartite network of neuron-gene association was created. Further a weighted, unipartite neuronal gene co-expression network (GCN) was created (Fig 3). GCN depicts the expression profile similarity among neuronal genes, where every neuron was represented by a node and the number of genes commonly expressed in any two neurons is represented as a weighted edge.

This gene co-expression network was further analysed and clustered using affinity propagation [18] clustering method in cytoscape [19] where affinity is based upon the shared number of genes between them.

### Gene ontological enrichment

The importance of these clusters was found out by statistically analysing phenotypic properties of each cluster. From the obtained clusters we figured out the unique genes *U*_*g*_ which are expressed in each cluster. These unique genes are then analysed on the basis of gene ontological enrichment studies using Biological Networks Gene Ontology tool (BiNGO) [20] to find out the unique contribution of each cluster in specific biological processes of the animal. Studies were performed to understand the uniqueness of specific genes expressed for each cluster on the basis of biological processes. Two types of gene ontological enrichment studies were performed to infer role of cluster in specific ontological processes. The first enrichment study of genes from each cluster was done against a background of all *C. elegans* genes, *G* (Eq (2)). In a more refined enrichment study, the genes from the clusters were enriched against a subset of *C. elegans* genes that are expressed only in neurons, *G*_*n*_ (Eq (3)).

$$\begin{array}{c} Gene\;enrichment\to U_{g}\subset G \end{array}$$ (2)

$$\begin{array}{c} Gene\;enrichment\to U_{g}\subset G_{n} \end{array}$$ (3)

### Essentiality of genes

With the objective of associating genes central to driver neurons, we linked essentiality of genes to those obtained from GO enriched genes belonging to three major clusters of GCN. Essentiality of genes was attributed with the help of ‘Database of essential genes’ [21, 22].

## Results

We treat *C. elegans* neuronal network as a complex adaptive system. *C. elegans* have evolved to have the present neuronal architecture by adapting to biotic and abiotic stresses over a long period of time. Driver neurons is a relatively new concept which is associated with a subset of neurons which when driven by an external input allows one to control the state of the whole network. In this paper, we aimed to identify driver neurons of *C. elegans* and to associate them with there phenotypic (phenoframe) as well as genotypic (genoframe) features. As part of phenoframe we have characterised driver neurons based on location, span and functional types. As part of genoframe we have identified the genetic underpinnings of the driver neurons.

Number of driver neurons are higher in *C. elegans* neuronal connectivity network (16.5%) as compared with its degree distribution conserved (9.6%) and random network (0.3%) models. Driver neurons tend to avoid hubs and have fewer synaptic connections (differential data in S1 Text and S1 Fig).

### Phenoframe of driver neurons

**Fig 4:**
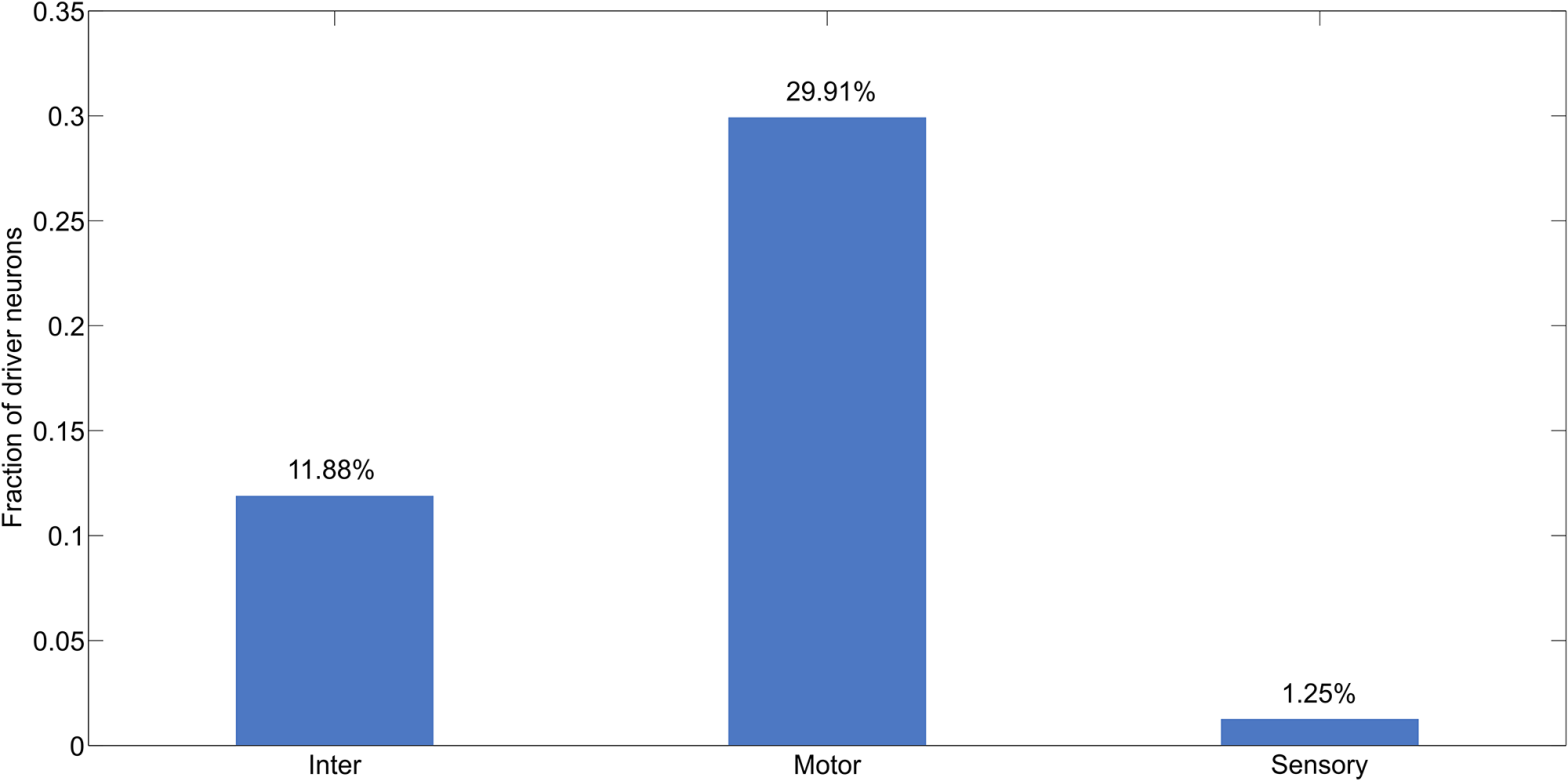
Distribution of driver neurons across different types of neurons: Sensory, Motor and Inter. The fraction of driver neurons was computed for each class separately.

*C. elegans* neuronal connectivity is fixed and does not change much over time. The notion of driver neurons implies control over the state of network with minimum extent of external inputs. While we are acutely aware that the exact implication of control of neuronal system is not clear, we believe that the notion of driver neurons offers us quantitative metrics of assessing control in such a simple neuronal system. Study of phenotypic properties of neurons led us to the observation that the largest proportion (29.91%) of the driver neurons identified with maximal matching algorithm were associated motor activities, followed by inter neurons (11.8%) and sensory neurons (1.25%) as shown in Fig 4. We therefore conclude that motor neurons are the primary means of achieving desired state of neuronal activity in the phenoframe of *C. elegans*. This is an interesting observation given that one might be tempted to hypothesise that sensory neurons are critical for driving the state of the neuronal network.

**Fig 5:**
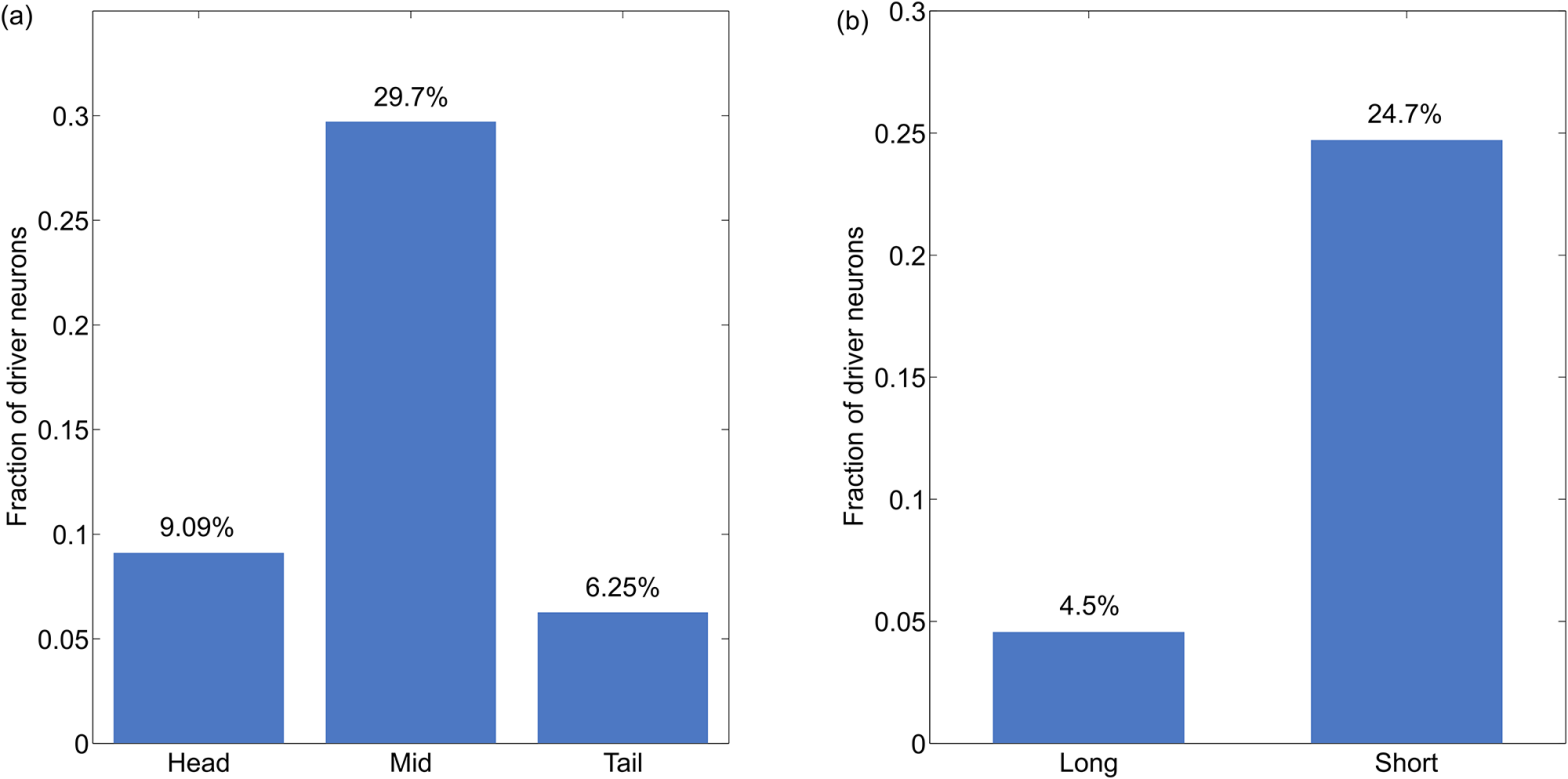
Distribution of driver neurons across other phenotypic features (location and span). (a) Location of neurons within the body of the organism. (b) Span of neurons in accordance with the length of axons.

**Fig 6:**
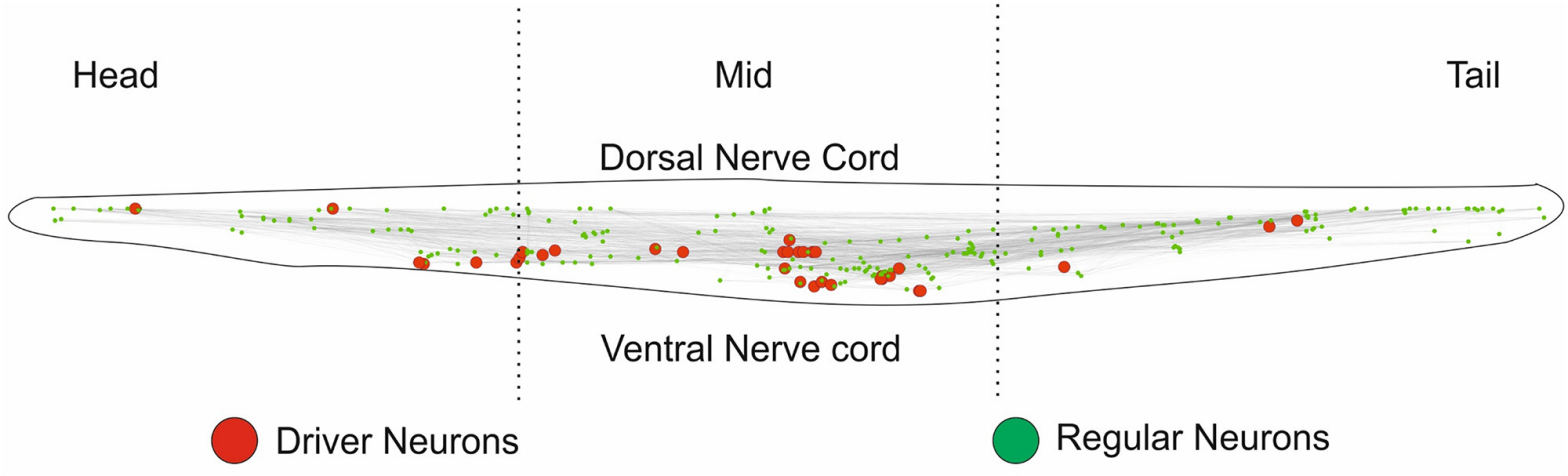
Visualization driver neurons of *C. elegans* and distribution of driver neurons across the body. The neurons are arranged in accordance with Cartesian coordinates presented within the body. This figure clearly shows presence of driver neurons in the mid-ventral region of the organism.

As part of phenoframe analysis we further observed association of driver neurons on the basis of location of neuron and their span as shown in Fig 5. Driver neurons are populated in the middle of the body (29.7%) whereas head and tail regions had sparse representation; 9.09% and 6.25% respectively (Fig 5(a)). Across different length spans of neurons, 24.7% of all the short spanned neurons were driver neurons. On the other hand, among the long span neurons only 4.55% were driver neurons as depicted in Fig 5(b). Interestingly, these phenotypic properties are also shared by VNC neurons that are known to control activities of bodily movements of animal. This could be interpreted to state that driver neurons which share phenotypic markers with VNC neurons could have evolved to control the movement of the animal thus changing its physical behaviour (Fig 6).

### Genoframe of driver neurons

**Fig 7:**
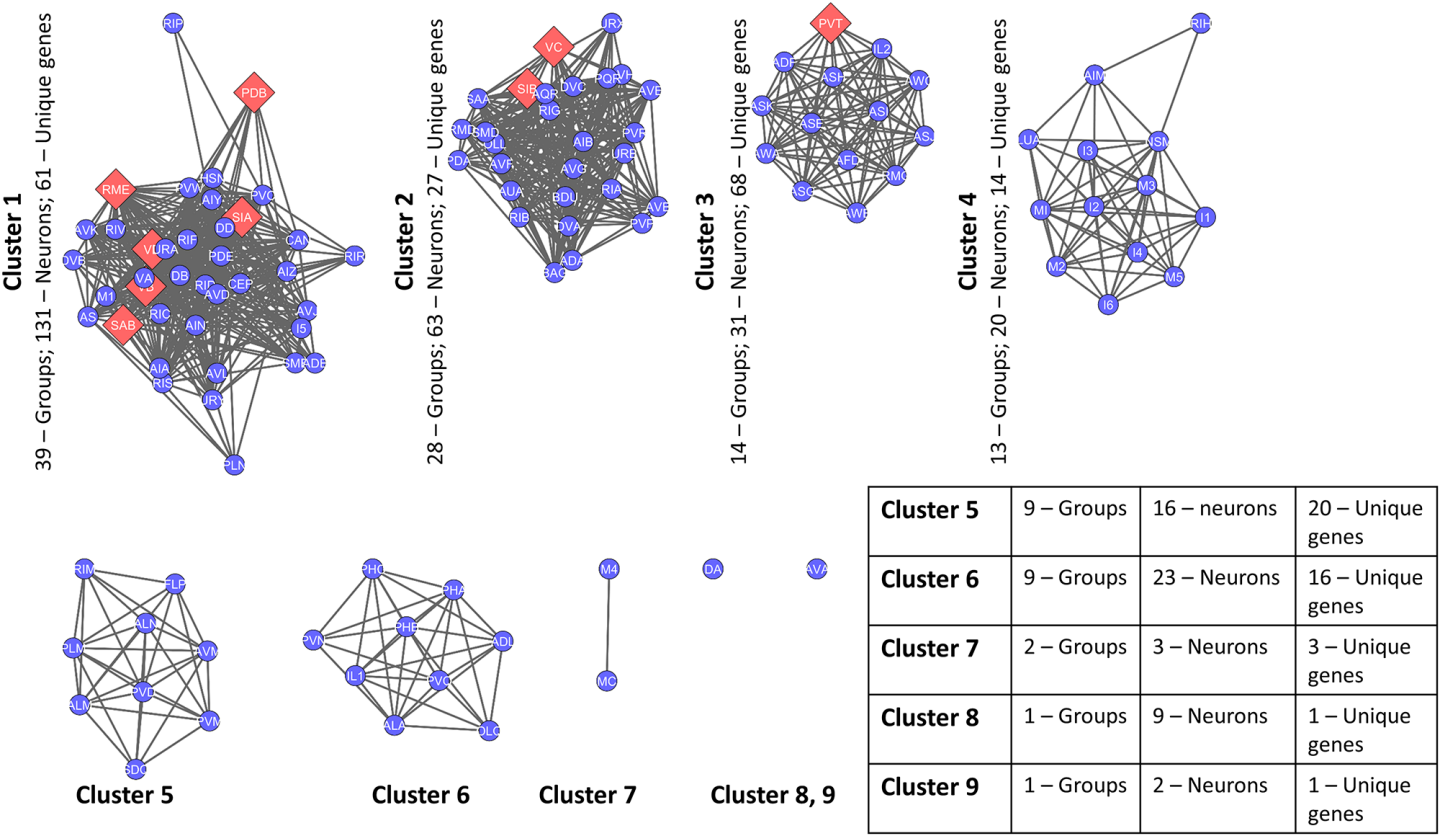
Nine neuronal groups (clusters) obtained starting from GCN using affinity propagation algorithm [18]. The three largest clusters contain driver nodes (red diamonds) hinting at possible role they play. Cluster three forms a perfect clique with highest number of unique genes expressed within a cluster.

Moving beyond phenotypic properties, we further explored the genetic association of driver neurons. Gene expression pattern of a cell plays a major role in specifying its biological function. We aimed to identify genetic correlates of driver neurons, named as genoframe. Starting from the gene co-expression network (Fig 3) we identified clusters of neurons with the assumption that neurons belonging to the same cluster could be associated with similar functions (Fig 7). We obtained 6 major clusters. Interestingly three largest clusters comprised of groups of driver neurons. Further we identified unique genes that each of the clusters is characterised with. Our intention was to obtain ontological features that characterise driver neurons as well as group of neurons that comprise of driver neurons. Phenoframe of clustered GCN can be found in S2 Text and S2 Fig.

#### Ontological studies of genes from driver neurons

We propose that the neuronal clusters containing driver neurons may play specific roles critical for evolutionary survival of the organism. To obtain ontologically relevant features, we performed gene ontological enrichment studies of the genes expressed in the clusters with driver neurons against the background of whole genome of *C. elegans*. We used three largest clusters with driver neurons for the purpose of GO enrichment studies.

We identified genes in each cluster that are unique (specifically expressed in a cluster) to it. We expect that significant ontological terms thus obtained may help us to identify biological processes and molecular functions central to the control. Gene ontological study showed that cluster 1 was mainly associated with (a) signalling process, (b) reproductive processes and (c) anatomical structural development of the animal, and could be relevant for controlling the movement of cells and its processes. Gene ontological studies performed for cluster 1 in the background of genes expressed exclusively in neuronal genes indicated an ontological association with reproduction and cellular localization. The ontological coordinates for cluster 2 and 3 are mainly concerned with biosynthetic processes. Thus we surmise that few of the most biological processes relevant to *C. elegans* are captured through the studies performed with clustering of GCN and gene ontological enrichment studies. Controlling driver neurons may have an implicit effect on cellular localization (p-value = 5.61 × 10^−4^) thus changing the anatomical structural development (p-value = 9.42 × 10^−4^) of the organism which in turn may have direct correlation with the state of the neuronal network. Another thing which came up in GO enrichment studies is the involvement of multi-organism reproductive processes (p-value = 6.94 × 10^−4^), which implies that these group of neurons can also control its reproductive behaviour which may have evolutionary impact.

### Genes ontologically relevant to driver neurons are not essential

Constructing a network of gene co-expression networks and clustering them facilitated gauging the role of genes that are critically related with driver neurons. Table 1 lists these genes for each of the three major clusters of GCN. None of the neuronal genes including those critical for driver neurons are biologically essential.

**Table 1:** List of gene obtained within unique set of genes after gene ontological enrichment of each clusters.

	Genes
**Cluster 1**	ldb-1, jnk-1, daf-10, syd-2, pll-1, syd-1, lin-14, gsa-1, hbl-1, tol-1, lat-1, unc-10, unc-47, ast-1, syd-2, cat-2, unc-40, hbl-1
**Cluster 2**	gcy-9, ets-5, gcy-33, gcy-31, gcy-32, gcy-34, gcy-25, fax-1, gcy-36, gcy-37, daf-16, gpa-8
**Cluster 3**	daf-11, gcy-6, gcy-5, gcy-4, gcy-3, gcy-14, gcy-8, odr-1, gcy-20, gcy-15, gcy-7, sma-6, gcy-22, gcy-23, gcy-19, sad-1, gcy-27, nhr-69, odr-7, skn-1, dsc-1, trx-1, gpa-5, egl-30, odr-3, gpa-4

## Discussion

Neurons play a very vital role in controlling the functional and bodily behaviour of the animal. In early parts of evolution neurons may have been associated with a specific function, but with time neurons may have started to decentralize functional tasks. The neuronal network of *C. elegans* is one such example where the body of ∼ 1000 cells is governed by ∼ 300 neurons. *C. elegans* neuronal network mapping is complete and hardly changes in its life-time. Similar to most other real world networks, this network is characterised with scale free topology, high average clustering coefficient ($\bar{C}=0.172$), and low characteristic path-length (*L* = 2.64), making it a good candidate for the study of controllability [23]. Other neuronal data sets such as cat cortical network and macaque brains cortex are partial and are known to be plastic in nature.

As compared to other measures of centrality, the unmatched nodes are shown to be efficient in controlling the network state using structural control theorem by Liu et. al. [5]. We utilised Hopcroft-Karp algorithm for finding unmatched nodes with computational complexity of $O(\sqrt{N}\times E)$[7], which makes it feasible to apply it on larger biological networked systems. Our results highlights the possible role of connectivity of neurons, as the number of driver neurons in degree distribution preserved model (9.6%) is close to that of *C. elegans* neuronal network (16.5%) in contrast of a random model (0.3%).

*C. elegans* neurons are divided into various classes on the basis of their association to function, position and length of the neurons. Finding a few important target neurons that are critical for achieving full control over the behaviour of the animal is an interesting task. The targets here are defined in terms of structural controllability and known as driver neurons. Driver neurons are further classified based on their phenotype and interestingly it is observed that a large number of such nodes are of motor type, and are located in the middle of the body with a short span. This gives us an insight into control mechanisms of the animal, thereby providing practical ways of controlling animal behaviour with the help of external stimulus. The phenoframe of *C. elegans* also suggests the presence of these neurons in ventral nerve cord of animal.

Gene ontological enrichment studies brings out unique function of genes in the background of housekeeping genes. By performing enrichment studies of specific groups of neurons with high number driver neurons we conclude that the *D*_*n*_ are primarily associated with reproductive behaviour of *C. elegans*, as compared to other functional associations. This may also imply that genetic make-up of regular neurons is largely concerned with growth and development of the organism. Driver neurons on the other hand are related to passing the genetic message to its progeny.

Thus targeting external signal towards the VNC can bring changes in the animal reproductive behaviour which in turn is speculated to have effect on the entire behavioural state of the animal. Further the importance of motor memory is also reflected with the fact that most of the driver neurons are involved in motor functions. This gives us a scope for controlling the behavioural state of the organism by stimulating motor neurons.

## Supporting Information

S1 Fig Driver nodes in real and random graphs. (a) Fraction of driver nodes as found in the *C. elegans* neuronal network (CeNN), and their corresponding random counterparts: Degree Distribution conserved model (DD) and Erdos-Reney graph (ER). (b) Fraction of driver neurons with low, medium and high degree in *C. elegans* neuronal network. These results are consistent with what is reported by Lui et. al [5].
(TIF)

S2 Fig Clustered GCN phenotypic distribution. Phenotypic distribution of neurons in the clusters of GCN in accordance with, (a) functional types, (b) location of the body, (c) Span of the neuronal axon.
(TIF)

S1 Text Driver nodes in real and random graphs. (PDF)

S2 Text Clustered GCN phenotypic distribution. (PDF)

S1 Data Neuron group gene expression profile. Neuron-gene bipartite graph is curated from WormWeb (www.wormweb.org). The data contains two columns: Column I—neuron group’s names and column II—genes expressed.
(XLSX)
