# Supplementary figures and images for: Control of Neuronal Network in Caenorhabditis elegans
Source: PLoS One. 2015 Sep 28;10(9):e0139204. doi: 10.1371/journal.pone.0139204 (PMC4586142; doi:10.1371/journal.pone.0139204)

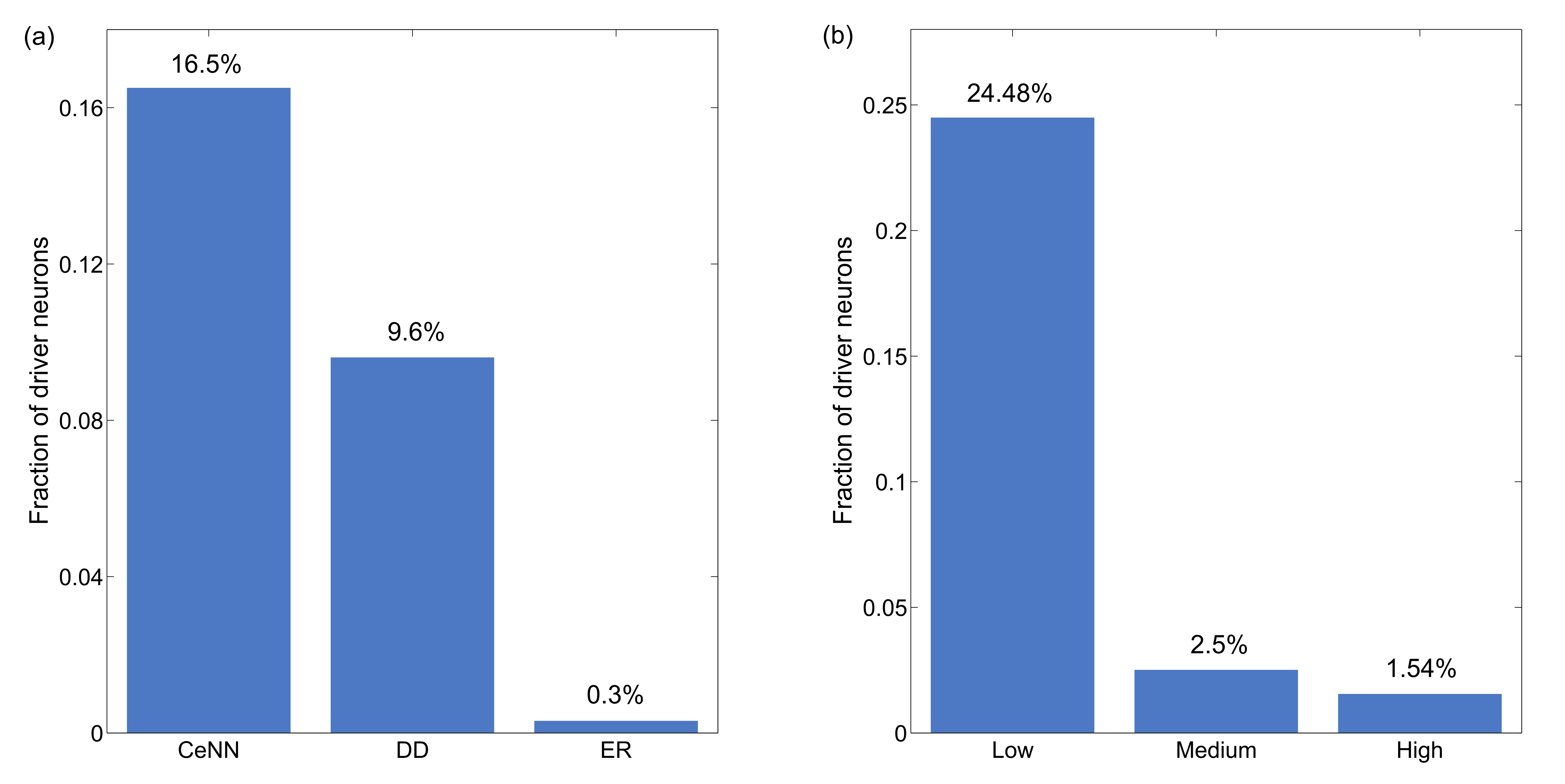

Supplement: S1 Fig — (a) Fraction of driver nodes as found in the C. elegans neuronal network (CeNN), and their corresponding random counterparts: Degree Distribution conserved model (DD) and Erdos-Reney graph (ER). (b) Fraction of driver neurons with low, medium and high degree in C. elegans neuronal network. These results are consistent with what is reported by Lui et. al [5]. (TIF) [file pone.0139204.s001.tif]

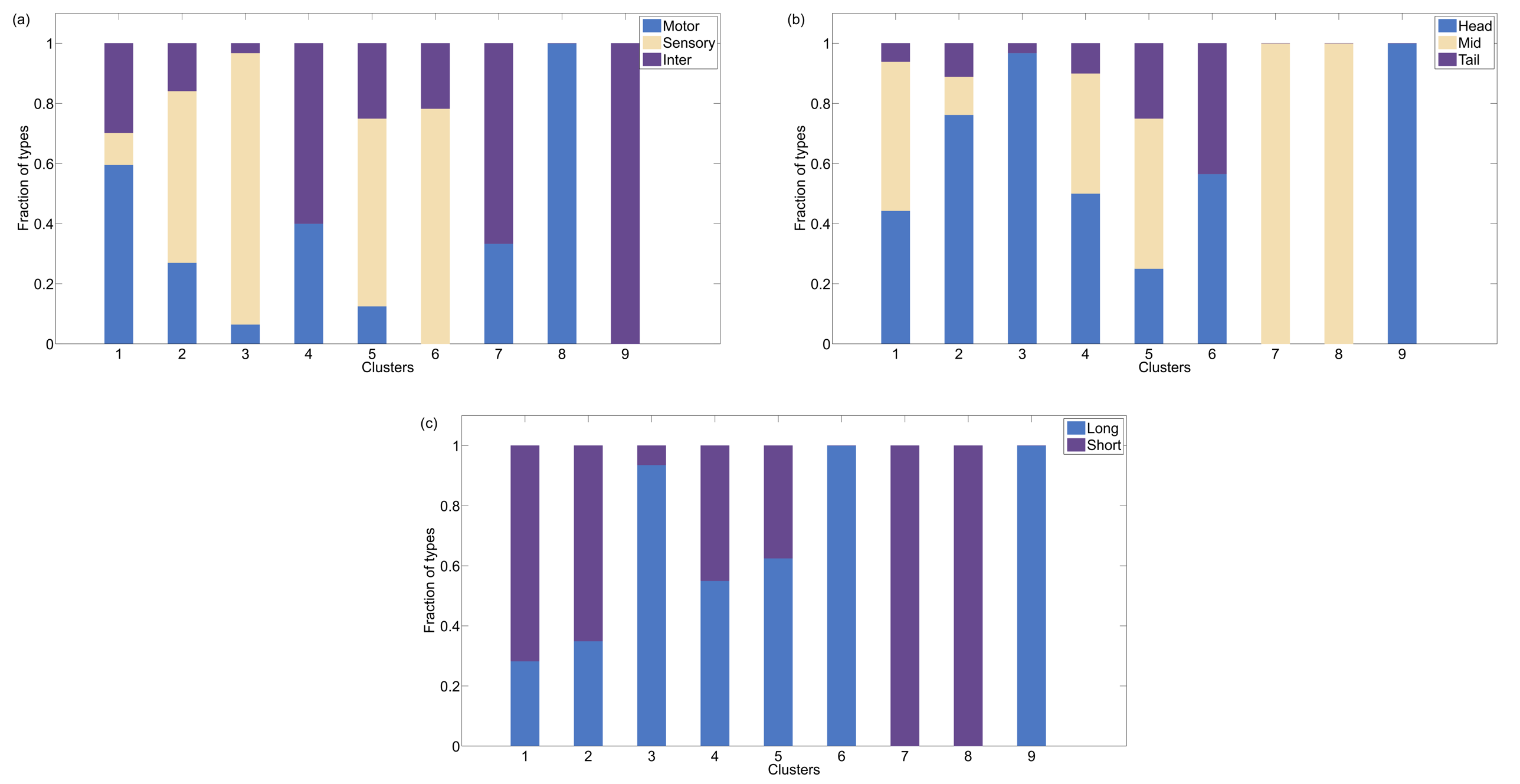

Supplement: S2 Fig — Phenotypic distribution of neurons in the clusters of GCN in accordance with, (a) functional types, (b) location of the body, (c) Span of the neuronal axon. (TIF) [file pone.0139204.s002.tif]
