# Supplementary material for: Control of Neuronal Network in Caenorhabditis elegans
Source: PLoS One. 2015 Sep 28;10(9):e0139204. doi: 10.1371/journal.pone.0139204 (PMC4586142; doi:10.1371/journal.pone.0139204)
Supplement: S1 Text — (PDF) [file pone.0139204.s003.pdf]

Driver nodes in real and random graphs

To fully control a network, minimum number of driver nodes required were calculated using the maximal matching criterion [1]. Matched nodes are the ones which share the link in a maximal matching, else they are unmatched. These unmatched nodes are of importance because these have directed paths to matched nodes allowing full controllability of the network. It had been seen earlier that the fraction of these nodes are higher in real world network as compared to its random counterparts [2]. For *C. elegans* neuronal connectivity network the fraction is much higher as compared to random counterpart but is partially achieved by making degree distribution conserved model of the network. Also, it has been observed that these nodes are lying in the lower degree(s) as shown in Fig S1 Fig.

References

1. Hopcroft JE, Karp RM. An $n^{5/2}$ Algorithm for Maximum Matchings in Bipartite Graphs. SIAM J Comput. Society for Industrial and Applied Mathematics; 1973;2: 225–231. doi:10.1137/0202019

2. Liu Y-Y, Slotine J-J, Barabási A-L. Controllability of complex networks. Nature. 2011;473: 167–73. doi:10.1038/nature10011
