# Supplementary material for: Control of Neuronal Network in Caenorhabditis elegans
Source: PLoS One. 2015 Sep 28;10(9):e0139204. doi: 10.1371/journal.pone.0139204 (PMC4586142; doi:10.1371/journal.pone.0139204)
Supplement: S2 Text — (PDF) [file pone.0139204.s004.pdf]

Clustered GCN phenotypic distribution

The GCN is composed of 116 neuron groups connected to each other via 4372 edges. This network is clustered using affinity propagation method in Cytoscape [1,2]. We obtained total 9 clusters out of which first 6 contains more than 95% of the neuron groups. Interestingly only three largest clusters comprised of groups of driver neurons. The cluster were analysed to see the type to neurons populations present in them on the basis of function, location and span. Fig S2 Fig shows the distribution of phenotypic properties of neuronal groups with in clusters.

The neuron groups in cluster 1 contains the highest number of driver neurons which are preceded by cluster 2 and 3 as shown in Fig 7. The phenotypic properties of these clusters (especially cluster 1) resembles that of driver neurons.

References

1. Morris JH, Apeltsin L, Newman AM, Baumbach J, Wittkop T, Su G, et al. clusterMaker: a multi-algorithm clustering plugin for Cytoscape. BMC Bioinformatics. 2011;12: 436. doi:10.1186/1471-2105-12-436

2. Smoot ME, Ono K, Ruscheinski J, Wang P-L, Ideker T. Cytoscape 2.8: new features for data integration and network visualization. Bioinformatics. 2011;27: 431–2. doi:10.1093/bioinformatics/btq675
